# Positive models of suffering and psychiatry

**DOI:** 10.1192/bjb.2023.104

**Published:** 2025-02

**Authors:** Ahmed Samei Huda

**Affiliations:** Pennine Care NHS Foundation Trust, UK

**Keywords:** Patients and service users, philosophy, psychosocial interventions, phenomenology, anthropology

## Abstract

Doctors’ typical reaction to patients’ suffering is to alleviate it when clinically appropriate. This has been described as a negative model of suffering, in contrast to the positive model of suffering. In the positive model, suffering can contain an important message of needed change, indicate a response to a psychosocial predicament or be a route to spiritual enlightenment. This approach is briefly critiqued, and circumstances where patients might prefer this approach are described. Doctors can work alongside professionals using this approach while also trying to alleviate suffering if indicated (such as if a patient wishes less suffering or if risk is involved).

An interesting article by James Davies that outlines the distinction between positive and negative models of suffering^1^ is accessible online (https://bulletin.hds.harvard.edu/the-rationalization-of-suffering/). This obviates the need to search for his papers from libraries or get copies of books to examine this discussion. As we swim in the medical sea, it can be difficult for doctors to encounter different viewpoints on how to respond to suffering other than the medical approach. This article provides an easily accessed description of an alternative model that could broaden our often narrow assumptions. The negative model is identified as being the typical medical approach, in which suffering is unpleasant with little intrinsic value and thus needs to be quashed. I will not focus on Davies’ criticism of psychiatric medication as similar to anaesthetics but will accept that doctors try to alleviate suffering, with the proviso that they apply clinical judgement as to when it is appropriate and usually as part of treatment goals agreed with the patient.^2^ Davies is a professional critic of psychiatry, but the focus of this article will be on working alongside a different model of helping people rather than demolishing it (although there will be some critique involved).

## The positive model of suffering

What is this positive model of suffering that is alien to conventional medical culture? It ‘holds that suffering can have a redemptive role to play in human life, that from affliction there can be derived some unexpected gain, new perspective, or beneficial alteration’.^1^ In the article, Davies leans heavily on Christian thinkers, but some of the important elements may be found in more secular concepts such as post-traumatic growth or hearing voices groups. Suffering is said to have potential benefits. The application of the negative model of suffering is said to be a failure of response to three particular aspects of suffering that are honoured or more appropriately responded to by the positive model of suffering. These include that suffering may contain an important message about making a needed change, that it might be a response to a grievous psychosocial situation, or that the sufferer may choose suffering as a pathway to spiritual transformation and enlightenment.

The model Davies describes seems less subtle than another Christian model of suffering which divides it into ‘essential suffering’ (an unavoidable suffering of human experience caused by life that can be offered solace by discussion with a preacher or therapist) and ‘inessential suffering’ (which can be avoided, for example, that caused by medical conditions for which seeking medical attention is recommended) as described by Luhrmann.^3^ I found this unconvincing, as to me it seems that an episode of psychosis is more like the inessential suffering of somatic illness than the essential suffering caused by the death of an infant child, even while accepting that mental illness is often more closely related to existential issues than somatic illness. Davies does not make this essential/inessential distinction explicitly, but it seems he regards mental health problems as essential suffering. An indicator of this is how he describes bipolar disorder in terms of the function it may have for someone instead of a recognition of its nature as a severe mood disorder.

Davies bases his positive model on a particular Christian model of suffering. Other religious traditions have different views on suffering. There are extensive Buddhist writings and thought about suffering which I cannot do justice to here even if the whole article were devoted to them. In Buddhism, suffering is regarded as often being caused by the ‘three poisons’ of greed, hatred/aversion and delusion/ignorance. Mindfulness interventions have been introduced into mental health with a rationale originally based on one element of the Eightfold Path to reduce suffering. This technique tries to redirect attention from dwelling guiltily on the past and worrying about the future towards focusing awareness on present action and experience in order to reduce anxiety and depression.^4^

## Critique of the positive model of suffering

Before discussing how psychiatrists can work with others who use the positive model of suffering to help people, we will address some weaknesses in the positive model.

The positive model of suffering as outlined by Davies is that suffering *can* have positive effects and that trying to ameliorate it can prevent these positive effects. But *can* is not the same as *always*. There is also the issue of who gets to decide whether the clinical priority is to reduce suffering or discern some message from it? What if the patient themselves wants to reduce suffering; does the clinician say ‘no, it may be good for you?’ In most cases, it's the patient who comes to the clinician for help, i.e. they are suffering and want relief and so consult the clinician, who then offers intervention. It is true they might not be consulting the clinician for relief of suffering; for instance, they might be requesting a fitness note or help in accessing benefits. Another possibility is that the patient presents with some other problem (for example, pain) and the clinician identifies the presence of another condition (such as depression) that the patient was either not aware of or did not wish help for. There also patients who do not regard themselves as suffering or consider that their suffering is not appropriate for medical attention.

From my own clinical experience, there are many cases where several assumptions of the positive model (for example, that a message is present that is discernible and useful) are not met. Of course, advocates may claim I simply did not look hard enough or recognise these messages from suffering, but it is hard to see what the valid useful message is from getting Alzheimer's disease or being sexually assaulted and experiencing post-traumatic stress disorder afterwards. Searching for the root cause or the hidden message may become the equivalent of hunting for the snark and at best waste the patient's time (and resources of whoever is paying for the intervention) if used as a sole alternative to medical care.

People have also claimed that there are messages behind general medical conditions. In the 1980s, during the AIDS epidemic, some proclaimed it was proof of a horrific homophobic message from God. This is something that Davies would condemn as an abuse of the positive model of suffering. The concept of identifying important psychosocial factors that caused and/or maintain the suffering can be addressed by the negative model of suffering in the clinical setting through the biopsychosocial formulation employed by psychiatrists, combined with appropriate interventions (family therapy or help with getting new housing and accessing benefits, for instance).^5^ Further, public health can address these causative and maintaining factors by direct interventions or influencing policy makers to address them.^6^ In other words, the negative model of suffering also addresses psychosocial factors causing suffering both on the individual and the social level, in contrast to Davies’ implication that it hinders pressure for change.

## Usefulness of the positive model of suffering

The most important questions regarding the positive model of suffering are not about whether it is true or not. It is better to ask: when is it useful, can it be used alongside medical approaches, and when can it be used without the typical medical response of alleviating suffering and reducing risk?

Doctors have a professional culture of aversion to suffering, and alleviating suffering is one of our core motivational values. Other professionals (including clerics and other religious professionals) and patients may have different priorities. I cannot be the only one to have encountered people who find the idea of a message or deeper purpose behind their suffering or events leading to suffering comforting to some degree. This may provide some sort of meaning regarding what has happened to them, for instance. ‘Everything happens for a reason’ is a commonly heard phrase reflecting many people's belief that there is a purpose behind negative experiences and attendant suffering.

Davies discusses how suffering may be a route to religious enlightenment. The issues of spiritual enlightenment are outside our professional knowledge base and competency as medical doctors. To my mind, they are better left to the clerics. I have seen people who have believed they are undergoing a spiritually enlightening experience that did not require treatment or other alleviation. Some people whose experiences were diagnosed as mental illness thought these were better described as ‘spiritual transformation’ instead,^7^ which indicates that they are not denying there may be suffering involved but consider that there is also enlightenment.

Davies describes how suffering may have a positive role, such as providing an important message about a needed change or indicating an important psychosocial predicament that should be addressed or embraced as a route to spiritual knowledge. This contrasts with medical aversion to suffering that must be alleviated as a priority. This positive model of suffering may not be true for all or for many cases that psychiatrists see. However, patients may find it useful to see their mental health problem as a sign that change is needed and comforting to think that there is a deeper meaning to their experiences. The positive model suggests suffering may indicate harmful socioeconomic predicaments that should be acknowledged, with the implication of these being acted upon. All medical specialties accept the importance of recognising and addressing the role of socioeconomic factors in causing health problems, so this is compatible with both positive and negative models of suffering. Some people diagnosed with mental illness regard it instead as a ‘dangerous gift’ or a ‘spiritual transformation’.^7^ Their conception of their experiences will mean that a way of working that does not focus on eliminating what the clinician regards as suffering but instead respects it as a meaningful experience and/or as a pathway to enlightenment will be more acceptable and regarded as more helpful.

There is an argument that the negative model of suffering is not just compatible with the positive model approach but is in fact helpful to or synergistic with the latter. A patient may be overcome by intense emotions or unusual experiences. Their capacity for action may be hindered by disorganisation or lack of motivation. These factors may greatly limit their ability to recognise or act on the messages for change that the positive model can identify. Medical treatment, by reducing the impact of these factors, can thus make it easier for the necessary changes to be identified and acted upon.

Some patients may request that only the positive model approach is used. Box 1 describes a list of steps that can aid doctors in deciding whether it is justifiable to step back from using the typical medical response to suffering while other professionals use the positive model approach.

**Box 1:** Guidance on using the positive model alone

Steps to help determine whether it is appropriate for the positive model to be used without the negative model of suffering
That the patient themselves prefers the positive model over relief of suffering of their own free will.
That the condition itself does not affect the patient's judgement such that they choose the positive model of suffering instead of alleviation of suffering.
That alleviating the suffering reduces the ability to recognise the need for change the ability to hear the need for change the motivation to change the ability to change and that this happens sufficiently frequently and to a degree that outweighs the likely benefits of alleviating suffering.
That using the positive model of suffering can be done within a reasonable time frame if we are choosing not to ameliorate the suffering, taking into account the patient's wishes, degree of distress, and any risk involved such as suicide or risk to others.

In step 1, care must be taken to ensure that this is the free choice of the patient and not a result of undue pressures from others, such as professionals with very negative views of medical interventions. Most clinical encounters are initiated by the patient, which would suggest that in these cases some relief of suffering is sought, although other valid patient motivations may be involved (see discussion above as to other reasons for seeing a clinician).

An example to illustrate step 2 is when a depressed patient believes that they deserve to suffer as a message from God about being Christendom's worst sinner and so reject treatment, but when the depression improves they no longer believe this and wish they had sought treatment earlier. Careful assessment as to the contribution of the condition to the belief may be required and, even then, the wrong conclusion may be drawn.

Step 3 may be hard to judge, especially by doctors, as we instinctively wish to alleviate suffering, but it is a justification for using the positive model approach instead of the medical approach to suffering. Step 4 is an important caveat of the risks of not using the negative model, including risks of harm to the patient and others and harm caused by waiting long periods to alleviate suffering. As long as there does not seem a good reason to intervene – such as risk of death – the doctor may consider adopting a watchful brief, being ready to offer alleviation of suffering if requested.

## Conclusion

Medical practitioners are used to working with other professionals with different models to them with the aim of helping patients with their clinical needs, even if these other models involve friction with or even hostility to medical models of care (and even if medical professionals’ tolerance for others’ models is not always reciprocated). Therefore, it is possible for psychiatrists to work alongside professionals using positive models of suffering to help people, even if, like Davies, they have inaccurate views of psychiatry and its treatments. The use of the positive model of suffering will need to be both with the consent of the patient and on the condition that they find it useful, given that it is not always true, and that it may be unhelpful to continually search for a helpful message that may prove elusive. People who regard their experiences as ‘dangerous gifts’ or ‘spiritual transformation’ often find the way doctors regard their experiences or try to intervene to alter them as incompatible with their wishes.^7^ They may find a positive model more helpful to them instead, but clinical judgement will still be needed if relevant, for example, if risk to self or others becomes a great concern.

## About the author

**Ahmed Samei Huda** is a consultant psychiatrist in the Tameside and Glossop Early Intervention Team, Pennine Care NHS Foundation Trust, Ashton-under-Lyne, UK.
